# The Effect of Chemical Threat Agents on Corneal Nerve Injury and Related Biomarkers

**DOI:** 10.51894/001c.164013

**Published:** 2026-07-31

**Authors:** Ashten Stambersky, Ebenezer Okoyeocha, Connor McGreer, Yusuf Mohammad, Sarah Marks, Steve Lundback, Neera Tewari-Singh

**Affiliations:** 1 College of Osteopathic Medicine, Michigan State University

## 12

### Introduction

Chloropicrin (CP) is a potent chemical threat agent (CTA) that causes severe ocular injury, with the cornea particularly vulnerable due to its anterior location and tear film reservoir. Exposure can result in photophobia, dry eye, corneal ulceration, scarring, and blindness. CP has been used in warfare and as an agricultural fumigant, posing ongoing risks. Currently, no effective treatments exist beyond symptomatic care. Phosgene Oxime (CX) is another highly toxic vesicant that penetrates tissues rapidly and carries high mortality. Although CP and CX are known to induce corneal inflammation and cell death, little is understood about their effects on corneal nerves. Corneal nerves are critical for tissue homeostasis, regeneration, and immune regulation. Objective: This study investigates corneal nerve morphology, function, and biomarkers of nerve injury following CP and CX exposure to better define mechanisms of injury and therapeutic targets.

### Methods

CX exposure was induced in C57BL/6 mice (5–9 weeks) using 10 μL applied for 15 or 30 seconds. CP exposure was performed in BALB/c mice using 2 μL at 2.5% or 10% for 1 minute. Sham and DMSO served as controls. Corneal sensitivity was measured using a Cochet-Bonnet aesthesiometer. Corneas were processed for flatmount or sectioning and stained with β3-tubulin to assess nerve density, substance P (SP) expression, nerve length, and branching. Gene expression of nerve injury and regeneration markers was quantified using qPCR and analyzed via the delta-delta CT method.

### Results

CX exposure significantly reduced nerve density at 30 seconds (24 hr). At day 1 post-CP exposure, 2.5% CP increased SP expression and varicosity co-localization compared to controls and 10% CP. By day 7, 2.5% CP showed greater nerve branching and length, whereas 10% CP caused more persistent damage. Corneal sensitivity decreased at both doses, but recovery occurred only in the 2.5% group. 10% CP at day 7 post-exposure showed significant increases in nerve injury biomarkers and decreases in nerve regeneration biomarkers.

### Conclusion

CP and CX induce dose-dependent corneal nerve injury, with higher doses impairing regeneration and function. Targeting nerve injury biomarkers may provide novel therapeutic strategies for CTA-induced ocular damage.

